# Mitigating acrylamide formation using agrifood waste materials: A concise review^☆^

**DOI:** 10.1016/j.fochx.2025.103308

**Published:** 2025-11-21

**Authors:** Mouandhe Imamou Hassani

**Affiliations:** Kaunas University of Technology, Department of Food Science and Technology, Radvilėnų pl. 19, Kaunas 50254, Lithuania; Institut National de Recherche Pour l'Agriculture, la Pêche et l'Environnement (INRAPE), Moroni, Comoros

**Keywords:** Acrylamide, Mitigation, Agrifood, Waste, And valorisation

## Abstract

Acrylamide is a toxic compound that occurs in thermally processed foods. Food waste valorisation is a common trend that alleviates the environmental burden by upcycling agricultural waste into higher-value materials. In this perspective, this review examined the potential of using agricultural waste to reduce acrylamide in food products, and the eventual challenges. Emphasis was placed on the mechanisms of mitigation of acrylamide using agrifood waste materials, recovery of valuable compounds from agrifood waste, practical applications in the food sector, potential challenges, and economic and environmental implications. Agrifood-sourced materials, such as antioxidants, dietary fibre (DF), pH modulators, and asparaginase, have been widely used to reduce acrylamide in heat-processed food products. Citrus waste is commonly used as a predominant material for acrylamide mitigation food products. Several factors such as concentration, composition of food and extract, and processing technique and conditions affect the efficiency of waste material in minimising and mitigating acrylamide formation.

## Highlights

• Agrifood waste offers sustainable strategies to mitigate acrylamide
• Key mitigators include antioxidants, pH modulators, dietary fibre, L-asparaginase
• Green technologies enable the extraction of compounds for acrylamide reduction
• Combining agrifood-based strategies with physical process is effective

## 1 Introduction

Acrylamide is a toxic compound that occurs in thermally processed foods. It is considered a major health hazard in the food industry due to its carcinogenic properties. A study revealed that female adolescents are more exposed to acrylamide than other population groups upon consumption of whole wheat bread, raising concerns for health risks associated with exposure to the processing contaminant (Lemos et al., 2024). In this regard, food and regional authorities such as EU, FDA, and Food Safety and Standards Authority of India (FSSAI) have established a benchmark for the permissible level of acrylamide in food products, which differs depending on the type of product. The Maillard reaction is the most common pathway that leads to acrylamide formation as it involves the reaction of free amino acids such as asparagine with reducing sugars. Consequently, N-glycoside is produced, which then undergoes various stages to produce melanoidin before the formation of acrylamide through decarboxylation of the Schiff base (Stadler et al., 2002). Processing conditions such as temperature, time, and pH are critical factors that affect the formation of acrylamide in food products (Stadler & Gökmen, 2024). Extensive research has been carried out since the discovery of acrylamide in 2002 to mitigate its formation, particularly in baked, fried, and roasted products. Several innovative approaches including cold atmospheric plasma blanching, microwave pre-thawing, product reformulation, enzymatic treatment, microbial treatment, ultrasound, irradiation, pulsed electric fields, high pressure processing, genetic engineering, vacuum treatment, and use of additives and plant extracts have demonstrated a great potential in reducing acrylamide in food products (Bachir, Haddarah, Sepulcre, & Pujola, 2022; Nateghi, Hosseini, & Fakheri, 2024; Peivasteh-Roudsari et al., 2024). It was also demonstrated that allicin,the organosulfur compound of garlic powder could be used as a key component in reducing acrylamide content during the acrylamide formation stage (Li et al., 2022). Low acrylamide content was recorded in Tritordeum bread due to the reduced content of asparagine, lower starch damage and denser bread structure as compared to common durum wheat bread (Wiwart, Szafrańska, & Suchowilska, 2025). This is portraying the significance of chemical composition, processing technologies, and techno functional properties in the reduction of acrylamide in foods. The selection of appropriate types of wheat flour, appropriate fermentation methods, and carefully managing baking conditions have been proposed as effective strategies to reduce acrylamide formation while preserving the quality of baked products (Marianelli et al., 2025). However, more sustainable approaches for mitigating acrylamide have been the subject of exhaustive investigation.

Food waste is an important issue within the food industry, posing considerable social, economic and environmental challenges by leading to unnecessary loss of resources throughout the food supply chain. The rapid growth of the human population requires an intense production of agricultural crops, which in turn leads to the generation of excessive waste materials. In fact, a reduction in food waste generates greater food security that extends the benefits to households (Wani et al., 2024). Food waste valorisation is a common trend that alleviates the environmental burden by upcycling agrofood waste into higher value materials. For instance, in the case of olive oil waste, new circular business models using innovative technologies, collaboration among farmers, businesses, and research common regulatory basis, and facilitating public financial measures are essential pathways towards economic, environmental, social, food and nutrition security dimensions (Donner et al., 2022). Ultrasound-assisted extraction of bioactive compounds from blueberry leaves using natural deep eutectic solvents demonstrated significant potential after optimization (Santos-Martín et al., 2023). In a biorefining attempt, grape stalks were initially fractionated, obtaining several product streams rich in polyphenols, hemicellulose, pectin, lignin, and cellulose before membrane treatment was used to recycle various materials within the process (Valle et al., 2025). Green alternatives have shown the efficacy of extraction / fractionation and selectivity of compounds from agrifood materials compared to conventional techniques.

The formulation of functional foods using food waste paved the way for various opportunities related to the use of agrifood waste. Despite the techno-functional challenges, this valorisation pathway is efficient, scalable, and tends to be economical. Agrifood waste is promoted by its antioxidant, antimicrobial, anti-inflammatory, and other bioactive properties. It was shown that pomegranate-peel extract supplementation could reduce acrylamide toxicity in rats due to its anti-inflammatory, anti-apoptotic, free radical scavenging and powerful antioxidant (Sayed et al., 2022). This mechanism of action is often utilized as a promising strategy for preventing the excessive formation of acrylamide in food products. More recently, several studies have been carried out on the application of food waste as a reformulation or pretreatment approach to reduce acrylamide in food products. The wide range of available waste types, such as press cakes, peels, pomace, etc., offer diverse uses in mitigating acrylamide as waste material can be used after extraction or in its raw form. This review examines the application of agrifood waste to reduce acrylamide in food products, and the potential challenges. The emphasis was placed on sustainable solutions and recommendations that will benefit researchers, industries, and policy makers from the food sector.

## 2 Overview of food waste materials and their valorisation

Food waste consists of edible and inedible food that occurs in various stages of the food supply chain from harvesting, processing, to consumption. Fruit and vegetable peels, seed hulls, pomace, and spent grain, and seed press cakes are the main waste streams generated by the agrifood industry. Due to their short shelf life, fruits and vegetables are difficult to manage and therefore result in a large waste content (Facchini, Silvestri, Digiesi, & Lucchese, 2023). According to the FAO report in 2019, a loss ranging from 3% to 18% occurred during the processing stage of vegetables and fruits with some exceptions (Facchini et al., 2023).

Circular economy has been recognized as a method for addressing unsustainable resource consumption and allowing companies to better understand the natural inputs that support them. In fact, waste valorisation offers greater insight into limiting a proportional amount of food waste through a variety of methods. The biorefinery concept was introduced to valorise all side streams of any supply chain into value-added products. It is worth mentioning that the treatment and processing technique applied to the waste material ultimately depends on the target end-use product. Moreover, the concept of waste valorisation constitutes an issue of dispute in terms of its nomination as agrifood waste material is often used as a feed product. In this regard, opinions differ among scientists, where it is argued insisted that the so-called agrifood waste and by-products should be rather entitled as low-value products instead of waste. Therefore, upcycling could be used as an adequate term for converting those agrifood materials into products with higher added value.

Depending on the source of the waste, macronutrients/micronutrients and a wide range of bioactive compounds are found abundant in various agrifood waste materials. DF from fruits and vegetable waste are excellent source of antioxidant and have demonstrated a potential to improve the functional properties of meat products when incorporated into them (Haque, Ahmad, Azad, Adnan, & Ashraf, 2023). Antioxidants from plant sources can be used as an effective therapeutic strategy against substances with high toxicity (Unsal, Cicek, & Sabancilar, 2021). Furthermore, food grade enzymes are often recovered from food waste, which suggests that the latter still contains natural enzymes that could mitigate the formation of toxic compounds. Due to the aforementioned reasons, it is reasonable for scientists to investigate the application of agrifood waste in reducing acrylamide in food products.

## 3 Mechanisms of acrylamide reduction using food waste materials

**Fig. 1:**
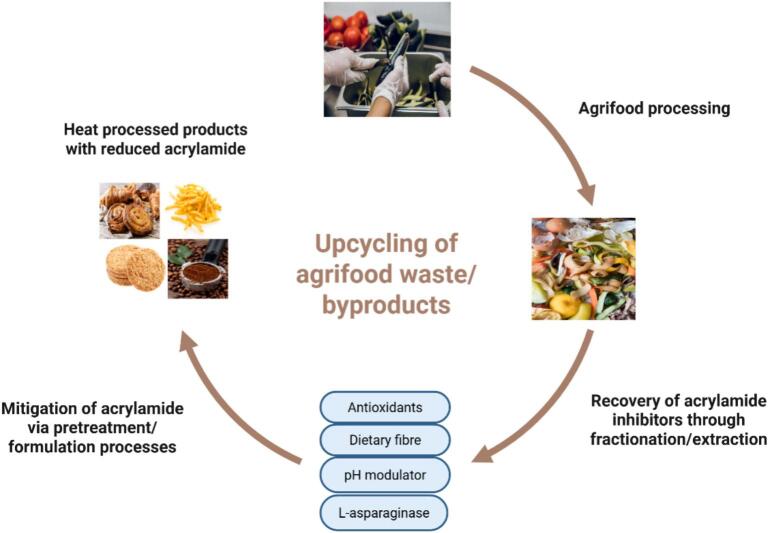
Upcycling of agrifood waste into acrylamide inhibitors in foodstuffs.

Fig. 1. shows a summary of various steps that lead to upcycling of agrifood waste/byproducts into acrylamide inhibitors in heat-processed foodstuffs. Antioxidants, DF, pH modulators and L-asparaginase are recovered from waste materials through various fractionation and extraction techniques, before application in heat processed products to reduce the acrylamide content.

### 3.1 Antioxidant activity

Among the different pathways by which chemicals are claimed to attenuate acrylamide formation, free radical scavenging capacity is exceptional, especially when using plant-derived antioxidants (Hamad et al., 2024a). In fact, a reduction in acrylamide content and improvement of sensory quality of potato chips occurred after soaking potato slices in natural antioxidant extract. (Assefa, Dessalegn, & Abegaz, 2024). The principal mechanisms of inhibition of acrylamide in food products were detailed by Zhang and Jin (2024): trapping of Maillard reaction intermediates by antioxidants at the C6 or C8 position of the A ring, and oxidation of unstableantioxidants to attack acrylamide.Additionally, polyphenols prevent the development of acrylamide in foods by inhibiting the oxidation of acrolein which is a common reactant that induces acrylamide formation (Baharinikoo, Chaichi, & Ganjali, 2022; Yang, Achaerandio, & Pujolà, 2016). Seyedi, Javanmarddakheli, Shekarabi, Shavandi, and Farhadi (2021) further summarized the reduction of acrylamide by phenolic compounds as the result of a combination of various mechanisms such as (1) radical scavenging activity, (2) carbonyl trapping effect, and (3) restriction of sugar degradation via the Maillard reaction. On the one hand, phenolic compounds such as gallic acid, ferulic acid, caffeic acid, (+)-catechin, and quercetin were found to inhibit acrylamide formation in bread matrices depending on the concentration (Mildner-Szkudlarz, Różańska, Piechowska, Waśkiewicz, & Zawirska-Wojtasiak, 2019). On the other hand, caffeic and gallic acids were tested in reducing acrylamide formation by Bassama, Brat, Bohuon, Boulanger, and Günata (2010) and Kotsiou, Tasioula-Margari, Capuano, and Fogliano (2011) in two independent investigations. Their results showed that the phenolic compounds were effective in the latter study, but ineffective in the former one. Various antioxidants can have opposing effects, as they can engage in different reactions during the Maillard reaction, leading to varying outcomes in terms of acrylamide formation and reduction (Zhang & Jin, 2024). The efficacy of phenolics in mitigating the formation likely depends not only on their radical-scavenging capacity, but also on their ability to interact with acrylamide precursors in the Maillard reaction, particularly free asparagine and reducing sugars. Factors such as the number and position of hydroxyl groups, the molecular weight, and the degree of polymerisation may also influence the reactivity and binding affinity of phenolics to acrylamide precursors. Therefore, in evaluating waste extracts for their potential ability to mitigate acrylamide, it is critical to go beyond classical antioxidant assays such as DPPH●, ABTS●, CUPRAC and/or ORAC. A more targeted investigation into the chemical structure, reactivity, and mechanism of action of individual phenolic compounds is necessary to identify effective inhibitors.

### 3.2 Dietary fibre

Waste materials rich in DF is also a viable approach to reducing acrylamide. DF is found abundantly in various waste sources and has been given great attention due to its essential physiological properties (Pathania & Kaur, 2022). Prior to application, common processing technique for fibre-rich materials is to soak them in water to eliminate free sugars, which could be termed as acrylamide precursors. For instance, the sugar-free apple pomace successfully mitigated the formation of acrylamide and enhanced nutritional properties in cookies due to its large amount of DF, mainly pectin (López-Ruiz et al., 2023). Apart from the action exhibited by phenolic compounds, the authors hypothesised that acrylamide formation could be limited by Michael addition due to the presence of nucleophile side chains of amino acids of proteins in water insoluble black currant and red cabbage (Zhang, Troise, Sun, & Fogliano, 2023). They also highlighted the low efficiency of commercial fibres in reducing acrylamide, thus promoting the higher value of waste material. López-Ruiz et al. (2023) evaluated the effect of unprocessed apple pomace that contained a high content of reducing sugars, especially fructose and as expected the formation of acrylamide increased by 386%. It is important to consider that the concentration of DF used is a partial replacement of the initial ingredient, which in turn results in a smaller amount of acrylamide precursors, such as asparagine and reducing sugars. In this regard, a more detailed analysis of sugar composition and a controlled examination of the Maillard reaction are needed to fully understand the mechanism behind the reduction of acrylamide (Wiwart et al., 2025).

### 3.3 pH modulation

Modulation of pH as a pretreatment technique is known to be effective in mitigating acrylamide generation in heat-processed foods. In this context, reducing the pH using organic acids can decrease the formation of acrylamide by protonating asparagine. This inhibits its nucleophilic interaction with carbonyl compounds, thus preventing the formation of a Schiff base, an essential intermediate in the Maillard reaction that leads to acrylamide production (Jung, Choi, & Ju, 2003; Medeiros Vinci, Mestdagh, & De Meulenaer, 2012). Acidic solutions such as citric, sodium pyrophosphate, and acetic acids have been reported to decrease the by up to 80% to 90% of acrylamide content (Huang et al., 2022). Lowering the pH appears to contribute to a reduction in acrylamide levels in whole breads, since the strong acidity of lemon juice (pH 2.70) brought the pH of the dough below 5.41—a threshold above which acrylamide formation typically occurs (Rwubatse et al., 2023). Decreasing pH using acids significantly inhibited acrylamide formation but enhanced 5-hydroxymethylfurfural (5-HMF) levels (Huang et al., 2022). In this case, the authors mentioned that pH regulators played a vital role in reducing acrylamide by enhancing the generation of methylglyoxal and glyoxal and promoted the formation of 5-HMF through the generation of 3-deoxyglucosone. 5-HMF does not pose an immediate health risk; however, it can be metabolized into 5-sulfoxymethylfurfural and 5-chloromethylfurfural in humans, substances that could potentially exhibit genotoxic and carcinogenic properties (Capuano & Fogliano, 2011). Therefore, it is necessary to look for new ways that will help mitigate both acrylamide and 5-HMF simultaneously.

### 3.4 Enzymatic approach

The use of asparaginase stands out as an acrylamide reduction strategy. L-asparaginase mitigates acrylamide formation by reducing its precursors by hydrolysing L-asparagine into aspartic acid and ammonia (Abedi, Mohammad Bagher Hashemi, & Ghiasi, 2023). The activity of L-asparaginase has no substantial influence on the qualitative attributes of the final products, such as alterations in colour or texture (Ciesarová & Kukurová, 2024). For example, the use of L-asparaginase had reduced acrylamide by 73% (Patial, Kumar, Joshi, Gupta, & Singh, 2022), 78% (Calabrese et al., 2024), 97% (Musa, Becker, Oellig, & Scherf, 2024b), and 68% (CarolinaVieira-Porto et al., 2024) in potato chips, homemade bread, oat cookies and roasted coffee, respectively. It is important to highlight the diversity and usefulness of L-asparaginase compared to other mitigation techniques in terms of efficiency, versatility, and flexibility. However, its application in the food industry is limited due to the cost of synthetic enzymes which ultimately leads to higher costs of food products. Agrifood waste seems to offer a solution to this problem, as it is a promising source of cheap enzyme production.

## 4 Extraction and fractionation

Before applications in food products, waste materials are generally processed or fractionated for the recovery of target functional materials. Bioactive compounds are some of the higher-value products that can be obtained from fruits, vegetables, cereals, and other food processing waste (Kumar, Yadav, Kumar, Vyas, & Dhaliwal, 2017). These compounds exert a strong potential against the formation and oxidation in food products, but also promote therapeutic values for human health (Banwo et al., 2021). Conventionally, methods such as Soxhlet extraction, maceration, infusion, decoction, percolation, hydrodistillation, and Osborne fractionation are used to recover valuable compounds from agrifood waste and by-products (More, Jambrak, & Arya, 2022; Yang, Dias, Pham, Barile, & de Moura Bell, 2024). However, more recently, ultrasound-assisted, enzyme-assisted, and pressurised liquid extractions, and many more, have emerged as green and sustainable technologies.

### 4.1 Extraction of antioxidants

#### 4.1.1 Enzyme-assisted extraction

Phenolic compounds, terpenes, saponins, and phytosterols are plant-based bioactive compounds with strong antioxidant capacity (Lizárraga-Velázquez et al., 2020). Achieving higher yield through process optimisation and mitigating negative environmental impact remain the principal driving forces for the development and applications of innovative, green, and sustainable extraction methods. Enzyme-assisted extraction (EAE) represents a promising alternative to conventional extraction since it does not require harmful solvents, nor higher processing temperature, which may result in degradation of the extracted biomolecules (Nadar, Rao, & Rathod, 2018). Shorter extraction time, recovery of targeted compounds, and reduced energy consumption are additional advantages of using EAE (Xiao et al., 2019). The main factors that affect EAE include temperature, pH, extraction time, enzyme concentration, type of enzyme, and substrate-to-solvent ratio. Extraction using viscozyme® L, cellulase and pectinase aided in recovering powerful antioxidants, mainly phenolic compounds from banana peel, with viscozyme® L being the most effective (Islam et al., 2023). In addition, the extraction of anthocyanins from aubergine peels was optimized with RSM (Amulya & ul Islam, 2023). It was shown that EAE of eggplant extract possessed higher yield and anthocyanin content than conventional extraction and also exhibited substantial phenolic content and antioxidant capacity (Amulya & ul Islam, 2023). The efficiency of cellulase was also confirmed by Nordin, Sulaiman, Noranizan, and Bakar (2023) at a treatment of 1.5% (*V*/V) enzyme concentration, a 1:4 g / ml solid to solvent ratio, an extraction temperature and a time of 50 °C and 120 min, respectively. In their research, cellulase treatment significantly increased the extraction of insoluble bound phenolics from MD2 pineapple peel by 99.79%. Currently, as mentioned earlier, the application of enzyme is limited because of high cost but novel production technologies using waste pave the way for cost effective utilisation of enzymes.

#### 4.1.2 Pressurised liquid extraction

The extraction of antioxidant compounds from classic and emerging waste can be also carried out by pressurised liquid extraction (PLE). PLE is described as a solid-liquid extraction performed at relatively high pressures (10–15 MPa) and high temperatures (50–200 °C)to operate above the boiling point of the solvent (Ramos, Kristenson, & Brinkman, 2002). Throughout the PLE process, high pressure and temperature keep the solvent in al liquid state for better efficacy (Rodriguez et al., 2023). Solvent penetration into the solid sample is facilitated by increasing solvent diffusion and the decrease of surface retention and viscosity, thus reducing the interaction of target compounds with the matrix and improving mass transfer (Lefebvre, Destandau, & Lesellier, 2021). PLE is a faster and greener alternative to conventional extraction techniques, especially when the solvent used is water and/or ethanol, which are labelled not only ecofriendly but also as food grade. Reduced solvent consumption and recovery of thermolabile plant antioxidants are prime characteristics ofPLE (Višnjevec, Barp, Lucci, & Moret, 2024). With the help of Response Surface Methodology (RSM), the PLE conditions were optimized to obtain anthocyanin-rich extracts from the black bean hulls (Teixeira et al., 2021). It was noticed that PLE showed higher potential than ultrasound and maceration methods in extracting anthocyanins. In another study, the PLE technique recovered a higher level of phenolics from pomegranate seed waste than high intensity focused ultrasounds (Guzmán-Lorite, Marina, & García, 2022). This highlights the importance of choosing a specific extraction method to recover specific target compounds from a particular plant source. Additionally, the extraction yield and composition of the PLE extract are influenced by the processing conditions. According to Rodriguez et al. (2023), the biological properties of the extracts, particularly the total phenolic content (TPC), are significantly affected by temperature. In fact, they observed that the highest TPC level was achieved at 120 °C, 9 ml.min^-1^ and 10 MPa. Such extracts can be incorporated directly into heat-processed food products or serve as pretreatment solution to mitigate acrylamide formation. To ensure a sustainable outcome, the solvent used for PLE may be green and food grade, such as ethanol and water.

#### 4.1.3 Ultrasound and microwave-assisted extraction

Ultrasound-assisted extraction (UAE) and microwave-assisted extraction (MAE) are green technologies commonly used to extract antioxidants due to their scalability. UAE functions at lower temperatures and requires shorter extraction times compared to conventional methods, helping to preserve heat-sensitive compounds while also reducing energy use (Garcia-Larez et al., 2024). In this method, the extraction of bioactive compounds from a matrix through the formation, expansion, and collapse of microbubbles is enhanced by sound waves ranging from sound waves ranging from 20 kHz to 100 MHz (Manousi, Sarakatsianos, & Samanidou, 2019). This phenomenon called cavitation leads to the generation of enough energy to disrupt the cell wall of the matrix, which in turn allows the release of targeted biomolecules (Bonifácio-Lopes, Teixeira, & Pintado, 2020). In this regard, time, temperature, frequency, amplitude, and solvent are considered as the major parameters that affect the extraction of antioxidants. UAE has been recommended for the industrial-scale recovery of polyphenols from spinach and orange waste, owing to its simplicity, cost-effectiveness, and other operational advantages (Montenegro-Landívar et al., 2021). Upon optimisation, the UAE of antioxidant bioactive compounds from rapeseed meal resulted in 99.30 mg GAE/g and 148.99 mg TE/g for TPC and antioxidant activity, respectively (Cisneros-Yupanqui et al., 2023). The usefulness of ultrasound has a huge impact on obtaining bioactive antioxidants from agricultural waste, hence replacing synthetic antioxidants. On the other hand, MAE has been extensively investigated for its potential to recover bioactive compounds from various agrifood residues and waste (Kumar et al., 2017). For example, extracts with high antioxidant activity were obtained from the cocoa bean shell using MAE (Mellinas, Jiménez, & Garrigós, 2020). The optimal extraction conditions were 12, 97 °C and 0.04 g/mL, for pH, temperature, and solvent ratio, respectively; with pH being the most influential parameter (Mellinas et al., 2020). As for MAE, nonionising electromagnetic radiations are used, with a frequency range of 300 MHz to 300 GHz, generate electric field by initiating ionic conduction, based on the electrophoretic transfer of ions and electrons (Nonglait & Gokhale, 2024). Due to the evaporation caused by the heating of water molecules,pressure is created within the plant cell followed by rupturing cell walls and facilitates the release of bioactive antioxidant compounds (Tapia-Quirós, Granados, Sentellas, & Saurina, 2024). When compared to maceration technique, MAE increased phenolic yield up to 45.70%,133.57%, and 65.30%, for broccoli leaves, florets, and stems, respectively (Rodríguez García & Raghavan, 2022). This study underlines the efficacy of MAE in biorefining all waste material derived from a specific agrifood industry.

#### 4.1.4 Supercritical fluid extraction

Owing to its economic feasibility, supercritical fluid extraction (SFE) is recommended for the vaporisation of residues from fruit waste (Restrepo-Serna & Cardona Alzate, 2022). Considering the recognition of supercritical fluids as a green technology, the application of SFE for the recovery of lipophilic compounds, including antioxidants, has been immensely reported (Vafaei, Rempel, Scanlon, Jones, & Eskin, 2022; Zhang et al., 2022). Generally, the most influential parameters during this extraction method are pressure, temperature, time, and flow rate. Upon optimisation of these parameters, the main advantages of SFE include superior purity of extracts, greater retention of bioactivity, and higher antioxidant yield of antioxidants (Vafaei et al., 2022). Additionally, the solvent to solid ratio, particle size, and percentage of modifiers are important operating variables (Tita, Navarrete, Martín, & Cocero, 2021). The SFE mechanism consists of separating the oil phase (extractant) from another plant matrix using inert gases pressured above the critical point where supercritical conditions are met (de Souza Correa et al., 2022). Supercritical fluids possess higher diffusivity and lower viscosity, which facilitates an increased penetration of the solvent through the solid matrix and, therefore, enhancing the extraction yields of a broad spectrum of biological compounds (Gallego, Bueno, & Herrero, 2019).

Aresta, Cotugno, De Vietro, Massari, and Zambonin (2020) developed an SFE protocol that helped recover bioactive compounds including trans-resveratrol, β-sitosterol, α-tocopherol (vitamin E) and ascorbic acid (vitamin C) from the wine making industry. Among all by-products, pomace was found to display the highest antioxidant activity. Furthermore, the extraction yield was significantly higher compared with traditional solid-liquid extraction procedure. This study illustrates the effectiveness of using SFE to biorefine various waste and by-products from a particular industry. In a different investigation, SFE-extracted total phenolic compounds and flavonoids from raspberries showed greater antioxidant activity when compared to previous studies (Velarde-Salcedo et al., 2023). Antioxidants recovered through SFE may be applied to heated products to reduce acrylamide; however, the techno-functional properties and safety of the final product deserve a very close attention. Taking into account their lipophilic properties, these antioxidants seem to be more suitable for food items with a high fat content.

When light is given to the above-discussed extraction methods, it can be observed that the extraction process, the source of extract, and the determination methods are important parameters in the final content of antioxidants. Hence, the need for optimising influential parameters for a cheap, rapid, and effective extraction of antioxidants from waste materials. Optimisation by using various tools is a common ground for all extraction techniques. In this regard, several bioactive compounds and antioxidants can be obtained efficiently using an integrated approach. For example, studies on the combination of MAE and natural deep eutectic solvents showed a potential to recover biomolecules from agrifood waste, with high extraction efficiency and antioxidant capacity (Tapia-Quirós et al., 2024).

### 4.2 Recovery of dietary fibre

Therapeutic values and techno functional properties are the major functional properties that are considered when DF is incorporated into food products. However, the potential to mitigate acrylamide has recently emerged as a new application of DF in food products. In particular, texture, shelf life, and freshness of heat-processed food products can be significantly increased by the incorporation of DF (Pathania & Kaur, 2022). A wide range of techniques are commonly used to extract DF from waste materials, including dry and wet grinding, various extraction methods (water, steam, acid, alkaline, ethanol, and acetone), enzymatic and enzymatic gravity approaches, homogenization, grinding and sieving, drying, solid-state fermentation, and hybrid methods that combine or modify these processes (Khanpit, Tajane, & Mandavgane, 2021). The maximum total separation of DF of 92.88% was achieved by alkaline extraction using sodium hydroxide from kiwi pomace (Wang, Li, Wang, Liu, & Ni, 2021). Furthermore, the DF of the Cat Chu mango peel was efficiently recovered using mild alkaline extraction, showing good physicochemical properties (Nguyen et al., 2024). To further improve the functional properties of DF, enzyme treatments are used, as was the case of DF recovered from broccoli by-products (Rivas et al., 2022). Another alternative to enzyme treatment is team explosion treatment. In fact, a steam pressure of 1.2 MPa, a residence period of 120 s, and a moisture content of 13% were the optimal parameters that led to the generation of a superior soluble DF from the residue of the Poria cocos peel (Wang et al., 2023). For ultrasound-assisted extraction of DF, optimal conditions were shown as liquid to solid ratio (49.8:1), temperature (26 °C), amplitude (38.6%) and treatment time (8.6 min) with the help of RSM (Kaur, Panesar, & Thakur, 2024). These conditions resulted in an extraction yield of 73.5%. This yield was further enhanced by using enzymatic treatment (α-amylase, protease, amyloglucosidase) after ultrasonication (Kaur et al., 2024). Rice bran is considered a source of functional soluble and insoluble fibres. Y-irradiation combined with enzymatic treatment resulted in soluble and insoluble DF with loose structure and improved physicochemical properties (Wei et al., 2024). The superiority of the conventional extraction method is obvious since at least two green technologies are commonly used to recover DF whereas alkaline extraction is mostly used alone. Exceptionally, during the extraction of DF from Queen pineapple waste, conventional alkaline extraction yielded 64.43% DF, whereas the UAE method yielded 86.67% after 22.35 min of sonication with a solid: liquid ratio of 27.5 g/ml and a ultrasonic amplitude (Dhar & Deka, 2023). The UAE significantly increased the capacity for water retention, glucose adsorption, and swelling and decreased the oil holding capacity (Dhar & Deka, 2023). In terms of techno-functional properties, ultrasound-assisted enzymatic extraction (UAEE) seems to offer a greater potential. In a study by (Panwar, Panesar, & Chopra, 2024), the extraction yields of DF from *Citrus limetta* peels were found to be 56.63% and 66.63% for alkaline extraction and UAEE, respectively. UAEE extracts exhibited higher thermal stability and crystalline nature than alkaline extracts. Depending on the source of the extract, an extraction method can demonstrate contradictory results. It can be concluded that the recovery of DF is influenced by several factors, such as extraction conditions, extraction method, and extraction source.

### 4.3 Production of L-asparaginase

Production of L-asparaginase requires the intervention of microorganisms and therefore the use of bioprocess engineering. Due to its abundant availability, agrifood waste is recognized as low-cost approach to produce L-asparaginase. The use of renewable waste materials that are abundant in sources of carbon and nitrogen presents a sustainable and economically viable strategy for the commercial production of L-asparaginase. This approach not only helps reduce production costs but also supports environmental conservation by repurposing agricultural and food industry by-products that would otherwise contribute to waste accumulation. For this purpose, solid-state, semi-solid-state submerged fermentation systems are the most common technologies employed (Lima Parente Fernandes, Alcântara Veríssimo, Cristina de Souza, Schwan, & Ribeiro Dias, 2021; Othman et al., 2022). The culture conditions (temperature, pH, incubation time and inoculum size) and the composition of the fermentation medium (waste) are the main factors that influence the production of L-asparaginase (Vimal & Kumar, 2022). To achieve cost-effective production, modern studies have utilized optimization tools such as Artificial Neural Network (ANN), RSM, and Path of Steepest Ascent (Ekpenyong et al., 2021; Sharma & Mishra, 2024). Rice husk, wheat bran, niger deoiled cake, maize bran, linseed de-oiled cake and pea pod husk were tested as substrates for the production and growth of L-asparaginase (Sharma & Mishra, 2022). After optimisation, the niger de-oiled cake resulted in the highest production yield. The authors emphasised that the ANN tool showed superior predictive capabilities even with a limited number of experiments (Sharma & Mishra, 2022).

## 5 Practical applications

Food products from various sectors are subject to acrylamide contamination and reduction according to regulations for an acceptable acrylamide concentration. This section deals with practical applications of the above-listed approaches for the mitigation of acrylamide in various food products. Table 1 summarises recent studies on reduction of acrylamide in foodstuffs using agri-food waste materials. Baking, frying, and roasting are the main cooking processes that generate a large amount of acrylamide content. Therefore, it is reasonable to explore the efficiency of agrifood waste-based antioxidants, DF, pH modulation, and asparaginase treatment in reducing acrylamide within products processed with these methods.

**Table 1:** Applications of Agri-Food-Based Approaches to Mitigation of Acrylamide in Food.

Matrix (food)	Mitigation strategy	Protocol	Key findings /Acrylamide reduction (%)	References
Potato Fried Chips	Antioxidant activity	The potato slices were soaked in potato, lemon, and pomegranate peels and olive leaves extracts before frying	86.11% and 69.66% for lemon peel and potato peels extracts, respectively	(Mohdaly et al., 2022)
Potato chip model	Antioxidant activity	Potato chips were immersed in grape pomce extract prior to frying	60.3%	(Xu et al., 2015)
Potato chips	Phenolic compounds	Potato slices were treated with orange peels before frying	Up to 44.8%	(Seyedi et al., 2021)
Rice-kofta	Antioxidant activity and pH modulation	Grapefruit and Guava Seed Extracts were incorporated in the formulation	Up to 40.70%	(Elsheshtawy et al., 2023)
Californian-style black olives	Antioxidant compounds	Extracts from waste and byproducts including alperujo (olive oil pomace), white skin grape, and orange peel were added to Californian-style black olives before the sterilization to reduce acrylamide	30%, 32%, and 12.9% for white skin, orange peel, and Alperujo extracts	(Fernández, Montero-Fernández, Pérez-Nevado, Martínez, & Martín-Vertedor, 2022)
Californian-style black olives	Antioxidant compounds	Extracts from waste and byproducts including alperujo (olive oil pomace), white skin grape, and orange peel were added to Californian-style black olives before the sterilization to reduce acrylamide	30%, 32%, and 12.9% for white skin, orange peel, and Alperujo extracts	(Fernández et al., 2022)
Biscuits	dietary fibre and antioxidant compounds	Wheat flour was partially substituted with chia seed fibre and flower waste in biscuits formulation	the addition of CS meal FRF and DCF waste powder may decrease the formation of acrylamide	(Sarkar et al., 2025)
Biscuits	Dietary fibre	Soluble dietary fibre (SDF) from tea residues was incorporated into the formulation of biscuits	SDFs together with bound polyphenols inhibited acrylamide formation in biscuits	(Ma et al., 2022)
Cookies	Dietary fibre	5% of the sugar-free lyophilised apple pomace (SRL) or 5% of sugar-free lyophilized and powdered apple pomace (SRLP) was enriched in cookies	62% for SL and 48% for SRLP	(López-Ruiz et al., 2023)
Potato crips	Dietary fibre	20% potato flake was substituted with water insoluble fractions of black currant pomace (IBC) and red cabbage waste (IRC) during formulation	33.7% and 85.3% for IBC and IRC, respectively	(Zhang et al., 2023)
French fries	Enzymatic approach	Raw potato slices were treated with flaxseed oilcake-based L-asparaginase before frying	Up to 93%	(Paul & Tiwary, 2020)
Mooncakes and fried potatoes	Enzymatic approach	Potato slices were treated with an agro-food-based L-asparaginase solution (10.12 mg ml − 1) for 45 min at 40 °C	52% and 86% for mooncakes and fried potatoes, respectively	(Gehlot, Kumar, & Pareek, 2022)
Live animal model *Caenorhabditis elegans*	Enzymatic approach	Onion and garlic peels were used as substrate to produce L-asparaginase which was tested in reducing acrylamide	The purified L-asparaginase demonstrated effect similar to asparaginase produced by *Bacillus licheniformis*	(Shakambari et al., 2017)
French fries and fried dough sticks	Enzymatic approach	Asparaginase was purified from soybean root nodules and tested for acrylamide reductions	Up to 90.08% and 87.90% on French fries and fried dough sticks, respectively	(Liu et al., 2019)

Reducing acrylamide in potato chips and French fries using antioxidant-rich extracts is dominant due to the popularity of these snacks and their health-related concerns. The potential of citrus waste demonstrated a reduction in acrylamide of 44.8% after soaking potatoes in orange peel extract before frying (Seyedi et al., 2021). Mohdaly, Roby, Sultan, Groß, and Smetanska (2022) studied the effect of extracts from olive leaves, pomegranate peels, lemon peels, and onion peels on the reduction of acrylamide formation in potato fried chips. They found that the waste extracts could hinder acrylamide formation by binding the precursors, namely asparaginase and reducing sugars, with the highest acrylamide reduction being the 86.11% lemon peel treatment. The authors underlined the crucial role that phenolic compounds, particularly naringenin, play. This study validates the principle of binding precursors as a prerequisite for an effective acrylamide mitigation. The level of acrylamide decreased by 60.3% after the concentration of polyphenol extract from Muscadine grape pomace increased by 0.1% (Xu et al., 2015). Although phenolic compounds often exhibit considerable antioxidant activity, the relationship between antioxidant potential and acrylamide inhibition seems complex. Xu et al. (2015) found no significant correlation between the antioxidant activity of phenolic-rich waste extracts and their effectiveness in reducing acrylamide levels. This finding suggests that higher antioxidant activity does not necessarily mean greater capacity to suppress acrylamide formation during thermal processing. Such inconsistency may be explained by differences in the structural and functional groups of antioxidants from various plant extracts. In addition, Spend coffee grounds had a significant impact in lowering acrylamide formation in whole wheat bread (Rwubatse et al., 2023). In that study, the authors drew a correlation between antioxidant capacity and acrylamide reduction, considering that the bread with the highest antioxidant activity was found to have the lowest acrylamide content. These studies suggest that the mitigation of acrylamide using agrifood waste depends not only on the concentration of waste-based extracts, but also on the specific phenolic compounds that induce antioxidant activity.

The incorporation of DF rich in phenolic compounds after removing monosaccharides is a robust approach, as they can play a double role of antioxidant DF in minimising acrylamide. Enrichment of fried potato crips with water-insoluble black currant pomace and red cabbage by-product successfully inhibited the accumulation of acrylamide (Zhang et al., 2023). Sarkar, Miah, Masum, Amin, and Alam (2025) partially substituted wheat flour with fibre-rich fractions from chia seed meal and dry chia flower waste powder for the preparation of biscuits. They observed an increase of acrylamide concentration in the substituted formulation when compared to the control sample. Since the noticed increase was below the benchmark, the authors claimed that a mitigating effect had been exhibited while preserving sensory attributes owing to the fibre-rich fractions. Another reason is that a previous study that utilized chia seed flour reported high levels of acrylamide content (1187 μg/kg) (Mesías, Holgado, Márquez-Ruiz, & Morales, 2016). Furthermore, pectin, a substance that can be recovered from agrifood waste, can be efficiently used to lower the pH of wheat dough due to its polymeric methyl de-esterified Galacturonic acid (Passos et al., 2018). In a study by Wang et al. (2022), pectin addition inhibited acrylamide by lowering the pH value in an Asparagine / glucose model system. Since acrylamide production peaks at pH 8, lowering the pH reduces the Maillard reaction and by extension lowers acrylamide formation (Vidhya et al., 2024). Nevertheless, enriching food products with DF is an unpopular approach to mitigating acrylamide formation even though the available studies have demonstrated its potential. This might be due to the risk of processing problems and unfavourable sensory properties in the product. In the case of biscuits, for example, enriching dough with fibre requires a higher water content, which can negatively affect biscuit quality by promoting the formation of the gluten network, ultimately leading to increased hardness (Villemejane, Roussel, Berland, Aymard, & Michon, 2013). Various factors should be considered during pretreatment or/and processing. Before considering the acrylamide reduction aspect of fibre in heat-processed products, it is necessary to perform preliminary studies on the water holding capacity and other techno-functional, and optimization of fibre concentrations. To promote this sustainable mitigation technique, more studies are needed to establish a solid knowledge base on the underlying mechanisms during the reduction of acrylamide content by DF, especially from waste materials.

Acids recovered from agrifood waste can be applied to heat-processed products in diverse forms to modulate the pH, which was demonstrated to limit acrylamide formation. Organic acids are commonly found in agricultural waste, especially citrus waste and by-products (Mamma & Christakopoulos, 2014). They have been widely used in the food industry for various purposes, including antioxidant, antimicrobial, and antimicrobial agents. Natural extracts are more effective due to their antioxidant potential and organic acids profile (Hamad et al., 2024b). It can be deducted that lowering pH might be showcased by fibre-rich and/or antioxidants-rich byproducts as demonstrated by Elsheshtawy, Salem, Elsabagh, and Sabeq (2023).

The production of L-asparaginase using agricultural waste is economically attractive due to the availability of wastes in agriculture-based countries (Mishra, 2006). Asparaginase may be generated by using waste including onion and garlic peelsas substrates (Shakambari, Sameer Kumar, Ashokkumar, & Varalakshmi, 2017) or by direct extraction and purification from byproducts such as soybean root nodules (Liu, Luo, & Lin, 2019). This is highlighting the variety of sources and pathways through which asparaginase can be produced for the purpose of mitigating acrylamide in foods. Agricultural waste was used as substrates to produce extracellular L-asparaginase from *Aspergillus terreus* sp. (Paul & Tiwary, 2020)*.* Asparaginase derived from this economically viable approach was combined with physical techniques, and as a result, nearly 93% of acrylamide reduction was achieved in French fries. This confirms the efficiency of the enzymatic approach in the reduction of acrylamide, as it was affirmed 10 years ago by (Palermo et al., 2016), who provided a qualitative science-based ranking of the mitigation strategies proposed in the FoodDrinkEurope Toolbox. In their evaluation, the authors underlined the easy handling of the enzyme and further predicted a considerable drop in the cost of the enzyme in the near future, which seems to be happening. It can be predicted that acrylamide mitigation with L-asparaginase will continue to gain popularity if the enzyme generation is cost-effective and sustainable. To achieve this goal, the use of agrifood waste materials along the production process is now inevitable.

It is worth exploring the impact of physical manipulations to reduce acrylamide, for example, an incubation temperature of 60 °C decreased acrylamide compared to 90 °C in wheat and rye cookies (Musa, Becker, Oellig, & Scherf, 2024a). It was also shown that the average occurrence of acrylamide could be reduced by lowering the temperature and frying with electric oven and less oil (Faraji, Shahidi, Shariatifar, & Ahmadi, 2024). Furthermore, the formation of acrylamide is influenced by cooking method, as oven and air fryer methods lowered acrylamide formation in French fries and sweet potato fries, respectively (Perestrelo et al., 2024). Freezing (<10 °C) was also demonstrated to decrease acrylamide level in potato chips and fries by improving cell disruption and leakage of reducing sugars and asparagine (Zhong et al., 2024). The formulation approach is mainly applied in bakery products to reduce the acrylamide content while antioxidant activity and pH modulation are often used in fried products. Hence, the importance of choosing the appropriate strategy to adapt in mitigating acrylamide content in various products from various food sectors. Several factors such as concentration, chemical composition, and processing technique and conditions significantly affect the efficiency of waste material in minimising and mitigating acrylamide formation.

## 6 Challenges and ways to overcome them

**Fig. 2:**
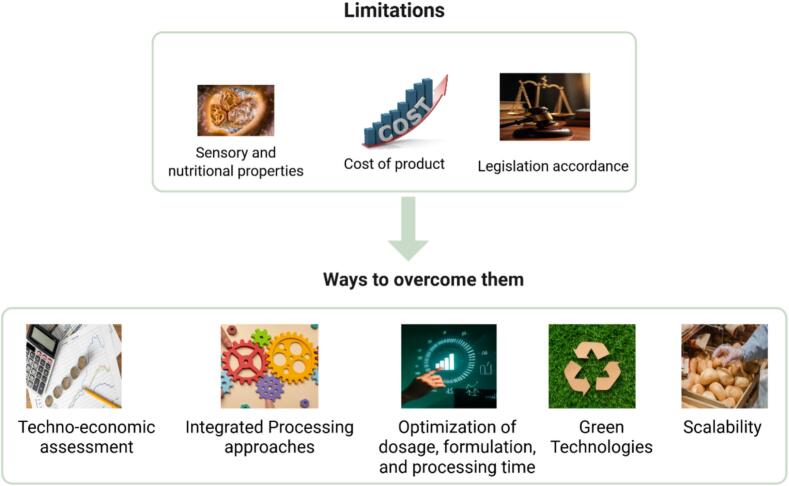
Limitations of using agrifood waste-derived materials in foodstuffs and potential solutions.

Food safety and food quality are crucial challenges when applying new concepts in food processing. While mitigating acrylamide in heat processed foodstuffs, it is necessary to ensure the safety of the product and avoid quality deterioration. It is important to recognise that each of the previously discussed above may face limitations in terms of their practical application within food production and industrial parameters. Factors such as compatibility with existing processes and formulations, impacts on sensory and nutritional qualities, regulatory approval, and cost can influence their feasibility (Nematollahi, Mollakhalili Meybodi, & Mousavi Khaneghah, 2021). Fig. 2. describes the main limitations related to the use of agrifood waste materials to reduce the acrylamide content in food products and their potential solutions. The physical and sensory properties of food can be altered while reducing the acrylamide content. The thyme, oregano, and garlic by-product could mitigate the acrylamide content in black table olives but scored lower values in the taste panel (Fernández et al., 2022). In an attempt to combine physical and chemical strategies to mitigate acrylamide in biscuits, Lo Faro, Salerno, Montevecchi, and Fava (2022) adopted an approach based on the FoodDrinkEurope Acrylamide Toolbox. The findings of this study indicated that colour indices and water activity exhibited the strongest correlation with acrylamide levels in biscuits. As such, these parameters have potential as reliable indicators for the monitoring and prediction of the acrylamide content during production processes. In contrast, another study affirmed that prediction of acrylamide based on colour is less reliable as the found a weak correlation between the colour of cookies and acrylamide (Musa et al., 2024a). This contradiction could be explained by the fact that the colour also depends on the type of flour and other ingredients used in the product formulation. Agrifood waste streams and by-products are known to contain persistent microbiological, chemical, and physical contaminants that contribute to existing food safety issues. Consequently, changes in the product quality and safety, shelf life, sensory properties, and consumer acceptance are expected. A correlation was drawn between sensory variables and toxic compounds studied including acrylamide and 5-hydroxymethylfurfural, as a decrease in acrylamide contend implied an increase in the sensory defect of roasted coffee (Cascos et al., 2024). Regardless of the mitigation strategy, optimising dosage, formulation, and processing time will contribute to preventing adverse effects on the functional properties of the end product. Moreover, implementing mitigation strategies through integrated approaches or pre- and posttreatment processes often leads to additional processing steps and longer production times, ultimately increasing overall processing costs (Maan et al., 2022). These added costs can influence the final price of the product and its competitiveness in the market. Consequently, a detailed cost analysis of both the process and the product is essential before implementing such strategies on a commercial scale.

Before applying the detailed strategies, a thorough risk analysis, risk assessment, and effective risk management must be carried out to avoid the formation of other toxic compounds (Onyeaka, Nwaiwu, Obileke, Miri, & Al-Sharify, 2023). As a result, according to Krzywonos, Difonzo, and Pasqualone (2025), it should be ensured that:

1. Safety Assurance - The absence of toxic substances, chemical or biological, originating from the waste materials used in extraction.
2. Green and Food-Grade Extraction – Capability to extract compounds using environmentally friendly and food-safe methods that avoid harmful solvents or reagents.
3. Industrial Scalability – Feasibility of applying the extraction process on a large industrial scale without compromising efficiency or cost-effectiveness.
4. Storage Stability – Retention of the functional properties of the extracted ingredients during storage, either in their natural form or when encapsulated or modified.
5. Functional efficacy - Demonstrated ability to enhance the nutritional or health value of the final food product, supported by biological or clinical evidence.
6. Nutrient bioaccessibility - minimal impact on the bioavailability of key nutrients in the food matrix.
7. Sensory compatibility - Little to no negative influence on the taste, texture, appearance or overall sensory quality of the final food product.

These criteria reflect the adherence of food safety and food quality standards while considering environmental sustainability. In addition, it is of utmost importance for authorities to guarantee consumers safety by launching specific laws and legislations that will regulate the suitability and safety of upcycled products (Socas-Rodríguez, Álvarez-Rivera, Valdés, Ibáñez, & Cifuentes, 2021). Establishing clear guidelines for assessing and managing food safety risks, along with ensuring that regulations comprehensively address the novel use of waste materials in agri-food systems, are essential for adopting a proactive approach. This does not only ensures public health, but also supports the advancement of circular economy principles within the food sector (Pearson, Mukherjee, Fattori, & Lipp, 2024).

## 7 Economic and environmental implications

An increase in the number of ingredients and processes in mitigating the acrylamide content in food products will ultimately affect production costs. In the strategies mentioned above, the financial implications are notably considered in the extraction, purification, recovery of high value products from agrifood waste and scalability. In the case of the enzymatic approach, for example, factors such as L-Asparaginase production costs and stability will influence the economic feasibility (Jana, Biswas, Ghosh, & Modak, 2025). Although data on cost assessment are scarce, the reduction of acrylamide in foodstuffs through waste valorisation, like all valorisation, requires a precise selection of materials, processes, and food products to minimise production expenses and time. In the case of the enzymatic approach, an economic analysis by (Jana et al., 2025) suggested that the cost effectiveness of L-asparaginase treatment is viable on an industrial scale with an increase of ∼4% in cost for enzyme-treated, acrylamide-free French fries. However, the innovative strategies described in this report to produce L-asparaginase would essentially lead to a more negligible cost increase due to the economic feasibility of agrifood waste-derived L-asparaginase. In light of that, to decrease acrylamide content in an economic and sustainable way, we suggest combining manipulation of physical factors which affect the acrylamide content, including cooking temperature and time, surface/area ratio, moisture content, and storage conditions in addition to agrifood waste-based approaches.

For businesses in the food and beverage sector, the practical implication is that waste-to-value products are well aimed at consumers who are environmentally conscious, health aware, and informed about issues such as acrylamide formation and food waste. Agrifood waste is used as feed, fertilizer, or often discarded. The conversion of agrifood waste into value-added products for acrylamide mitigation also alleviates the environmental burden of such materials. However, it is a complicated process due to the extraction process's inefficiency and the high organic content (Atalar et al., 2025). The economic implication and environmental implications go hand in hand as for the strategies to succeed in the market, as consumers who show environment awareness are more likely to accept food products based on agrifood waste. Valorisation and upcycling through the detailed approaches will ultimately reduce waste but also use resources efficiently in reducing acrylamide content in heat processed products.

## 8 Conclusions and future recommendations

This review underscores the potential of agrifood waste for the mitigation of acrylamide while promoting both environmental sustainability and economic prosperity. Antioxidant activity, pH modulation, incorporation of dietary fibre, and L-asparaginase addition are the main pathways based on agrifood waste to reduce acrylamide in heat proceeds food products in heat. Green technologies are utilized to fractionate waste and extract safe and functional value-added materials. The main driving force is the low cost and availability of agrifood waste, in addition to their sustainable valorisation. To reduce acrylamide content in an economic and sustainable way, this comprehensive analysis suggests combining manipulation of physical factors that affect acrylamide content such as cooking temperature and time, surface/area ratio, moisture content and storage conditions in addition to agrifood waste-based approaches. More studies are needed to explain the mechanisms of waste on the reduction of acrylamide. For scalability, life cycle assessment and techno-economic evaluation of the process/product needs to be carefully performed.

## CRediT authorship contribution statement

**Mouandhe Imamou Hassani:** Writing – review & editing, Writing – original draft, Visualization, Validation, Supervision, Software, Resources, Project administration, Methodology, Investigation, Funding acquisition, Formal analysis, Data curation, Conceptualization.

## Declaration of generative AI and AI-assisted technologies in the writing process

During the preparation of this work, the author used Writefull and Quilbot AI in order to improve the grammar of the manuscript. After using this tool/service, the author reviewed and edited the content as needed and takes full responsibility for the content of the publication.

## Declaration of competing interest

The authors declare that they have no known competing financial interests or personal relationships that could have appeared to influence the work reported in this paper.

## Data Availability

No data was used for the research described in the article.
